# N-terminal pro-brain natriuretic peptide reflects both left ventricular diastolic dysfunction and myeloma-related renal insufficiency and robustly predicts mortality in patients with symptomatic multiple myeloma

**DOI:** 10.18632/oncotarget.26647

**Published:** 2019-02-01

**Authors:** Yoshiaki Abe, Tetsuya Kobayashi, Yoshiaki Usui, Kentaro Narita, Hiroki Kobayashi, Akihiro Kitadate, Daisuke Miura, Masami Takeuchi, Kosei Matsue

**Affiliations:** ^1^ Division of Hematology/Oncology, Department of Internal Medicine, Kameda Medical Center, Chiba, Japan; ^2^ Division of Cardiology, Kameda Medical Center, Chiba, Japan; ^3^ Division of Cancer Information and Control, Department of Preventive Medicine, Aichi Cancer Centre Research Institute, Aichi, Japan

**Keywords:** diastolic dysfunction, frailty, multiple myeloma, N-terminal pro-brain natriuretic peptide, prognosis

## Abstract

We retrospectively explored the prognostic relevance of N-terminal pro-brain natriuretic peptide (NT-proBNP) and the association of NT-proBNP with cardiac and renal functions in 153 patients with newly diagnosed symptomatic multiple myeloma and no concomitant light chain amyloidosis who received novel agents. We also examined the usefulness of the new frailty system recently introduced by Mayo Clinic (combining age, performance status, and NT-proBNP). Patients with higher NT-proBNP levels (≥300 ng/L) had a significantly higher incidence of left ventricular diastolic dysfunction (LVDD) and myeloma-related renal insufficiency and significantly shorter overall survival (OS) than did those with lower NT-proBNP levels (<300 ng/L). NT-proBNP remained predictive of OS on multivariate analysis. Mayo Clinic's new frailty system showed excellent discrimination of OS. Furthermore, the Instrumental Activity of Daily Living (IADL) score (not evaluated in Mayo Clinic's study) predicted OS independently of this system, and a sharper discrimination of OS curves was obtained by the incorporation of IADL into this system. Our findings demonstrated that NT-proBNP levels were associated with both LVDD (as a host risk factor) and myeloma-related renal insufficiency (resulting from the disease aggressiveness) and provided predictive information regarding OS in patients with symptomatic myeloma. Furthermore, we, for the first time, validated Mayo Clinic's new frailty system. Our modification further improved Mayo Clinic's system by newly incorporating the IADL score.

## INTRODUCTION

The clinical outcome in patients with symptomatic multiple myeloma (MM) varies depending on intricate interactions between biological properties of the plasma cell clone and host factors [1]. Several prognostic systems have been developed and validated using clinical trials and real-world cohorts [2–5]. Among them, the International Staging System (ISS) and the revised (R)-ISS are the most representative: both systems predominantly include information associated with tumor burden and biological features of myeloma [3, 4]. Meanwhile, several recent studies have shown that patient frailty had an independent influence on mortality in the context of these prognostic systems [6–10]. The International Myeloma Working Group (IMWG) proposed a geriatric assessment including age, Katz Activity of Daily Living (ADL), the Lawton Instrumental ADL (IADL), and Charlson Comorbidity Index (CCI), which significantly predicted mortality and the risk of treatment toxicity [8]. Thus, frailty assessment has gained more attention in MM prognostication.

The N-terminal fragments of the prohormone brain natriuretic peptide (NT-proBNP) are secreted predominantly from the left ventricle in response to high ventricular filling pressures and wall stress [11–13]. In addition, plasma NT-proBNP concentrations are strongly influenced by renal function. These well-known facts allowed us to speculate that these biomarkers may capture not only the aggressiveness of myeloma but also the host factors, including frailty and tolerability against chemotherapy, and consequently, effectively predict survival. Milani *et al*. demonstrated the relationship between NT-proBNP levels and patient frailty, delineated by age, Eastern Cooperative Oncology Group performance status (ECOG-PS), ADL, and CCI, further showing the prognostic implications of NT-proBNP in patients with MM using a cohort from Mayo Clinic [14]. They also proposed a new frailty system (combining age, ECOG-PS, and NT-proBNP) as a useful stratification system for MM. However, their cohort included relatively younger patients (median: 65 years) compared to those observed in real-world settings (median: >70 years) [15]. Therefore, validation using an independent cohort including real-world population has been needed. In addition, no study has elucidated what the NT-proBNP levels directly reflect in frail myeloma patients or in patients ultimately with risk for all-cause mortality. NT-proBNP has been demonstrated to reflect cardiac dysfunction in heart failure patients [16, 17]; however, MM patients frequently present renal insufficiency, which is unrelated to cardiac functions, and thus, might possibly undermine the performance of NT-proBNP for the evaluation of cardiac function in this population. In this study, we aimed to elucidate the clinical behaviors and usefulness of NT-proBNP in patients with symptomatic MM by identifying clinical factors and mechanisms that are reflected by NT-proBNP, which more directly affect mortality, and investigating the applicability of the prognostication using NT-proBNP and Mayo Clinic's new frailty system to our real-world cohort.

## RESULTS

### Demographic and baseline characteristics of patients

Baseline clinical characteristics of patients are shown in Table 1. The median age of the patients was 74.2 (interquartile range [IQR]: 66.7–80.8) years. The median observation period for all patients was 26.4 (IQR: 10.8–49.1) months. Bortezomib and lenalidomide were used in 148 (96.7%) and 136 (88.9%) patients, respectively. The median NT-proBNP levels were 309 (IQR: 126–1061) ng/L. Echocardiographic data for the evaluation of left ventricular (LV) diastolic function was available in 131 (85.6%) patients (routine work-up for diastolic function evaluation was initiated in 2012 at our institution): these patients had no differences in baseline clinical parameters compared with the remaining patients (Supplementary Table 1). Left ventricular diastolic dysfunction (LVDD) was detected in 49.6% of evaluable patients.

**Table 1:** Baseline clinical characteristics of all patients and comparison between patients with lower and higher NT-proBNP levels

Clinical factors	All patients	NT-proBNP levels		*P*
	*n* = 153	Lower (< 300 ng/L) *n* = 75	Higher (≥ 300 ng/L) *n* = 78	
Observation period, months (median [IQR])	26.4 [10.8, 49.1]	32.8 [15.4, 53.6]	20.4 [10.2, 42.7]	0.058
**Background and/or frailty parameters**				
Age, years (median [IQR])	74.2 [66.7, 80.8]	70.7 [65.1, 78.1]	75.9 [70.2, 81.2]	0.020
Sex, male (%)	76 (49.7)	39 (52.0)	37 (47.4)	0.62
ECOG-PS, ≥2 (%)	83 (54.2)	31 (41.3)	52 (66.7)	0.002
IADL, ≤4 (%)	72 (47.1)	26 (34.7)	46 (59.0)	0.003
CCI, ≥2 (%)	74 (48.4)	22 (29.3)	52 (66.7)	<0.001
**Past medical histories**				
Coronary artery disease (%)	11 (7.2)	4 (5.3)	7 (9.0)	0.53
Arrhythmia (%)	10 (6.5)	1 (1.3)	9 (11.5)	0.018
Chronic heart failure (%)	15 (9.8)	1 (1.3)	14 (17.9)	0.001
Hypertension (%)	86 (56.2)	38 (50.7)	48 (61.5)	0.19
Diabetes mellitus (%)	34 (22.2)	15 (20.0)	19 (24.4)	0.56
**Myeloma-related factors**				
Heavy chain type, IgG (%)	79 (51.6)	48 (64.0)	31 (39.7)	0.004
Light chain only myeloma (%)	32 (20.9)	10 (13.3)	22 (28.2)	0.029
Albumin, g/dL (median [IQR])	3.4 [2.8, 3.9]	3.7 [3.2, 4.0]	3.1 [2.5, 3.6]	<0.001
Beta 2-microglobulin, mg/L (median [IQR])	4.6 [2.9, 8.3]	3.0 [2.3, 4.6]	7.7 [4.5, 13.9]	<0.001
eGFR, mL/min/1.73 m^2^ (median [IQR])	50.3 [27.8, 69.0]	64.8 [50.3, 74.6]	33.9 [13.3, 50.5]	<0.001
Hemoglobin, g/dL (median [IQR])	9.6 [8.4, 11.4]	10.2 [9.2, 12.2]	9.1 [7.8, 10.2]	<0.001
Corrected calcium, mg/dL (median [IQR])	9.7 [9.2, 10.4]	9.4 [9.1, 9.9]	9.9 [9.5, 11.0]	<0.001
Hypercalcemia-associated renal insufficiency (%)	15 (9.8)	1 (1.3)	14 (17.9)	0.001
Involved FLC, mg/L (median [IQR])	541 [128, 3225]	360 [98, 813]	2330 [197, 8450]	<0.001
Light chain cast nephropathy (%)	35 (22.9)	3 (4.0)	32 (41.0)	<0.001
Myeloma-related renal insufficiency (%)	41 (26.8)	4 (5.3)	37 (47.4)	<0.001
LDH, high (%)	41 (26.8)	14 (18.7)	27 (34.6)	0.029
High-risk CA (%)	31 (20.3)	15 (20.0)	16 (20.5)	1.0
R-ISS, ≥ stage II (%)	126 (82.4)	52 (69.3)	74 (94.9)	<0.001
DS system, stage III (%)	93 (60.8)	32 (42.7)	61 (78.2)	<0.001
Use of new agents (%)	153 (100)	75 (100)	78 (100)	1.0
Bortezomib use (%)	148 (96.7)	73 (97.3)	75 (96.2)	1.0
Lenalidomide use (%)	136 (88.9)	69 (92.0)	67 (85.9)	0.31
ASCT recipients (%)	43 (28.1)	26 (34.7)	17 (21.8)	0.11
**Cardiological parameters**				
NT-proBNP, ng/L (median [IQR])	309 [126, 1061]	126 [65, 195]	1058 [450, 2901]	<0.001
LVEF, % (median [IQR])	70.0 [66.2, 73.0]	70.0 [67.0, 72.0]	70.0 [65.7, 73.2]	0.90
LVDD (%)^†^	65 (49.6)	22 (32.8)	43 (67.2)	<0.001
E/e’ (median [IQR])^†^	12.6 [10.0, 15.4]	11.3 [9.5, 12.9]	14.3 [12.4, 16.6]	<0.001
LAVI, mL/m^2^ (median [IQR])^†^	43.7 [33.2, 51.4]	40.3 [30.4, 48.5]	47.5 [37.4, 59.4]	0.001
Septal e’, cm/s (median [IQR])^†^	5.7 [4.8, 7.1]	5.8 [5.3, 7.1]	5.6 [4.3, 7.0]	0.20
TR velocity, m/s (median [IQR])^†^	2.60 [2.33, 2.83]	2.4 [2.2, 2.6]	2.8 [2.6, 3.1]	<0.001

Abbreviations: ASCT, autologous stem cell transplantation; CA, cytogenetic abnormality; CCI, Charlson Comorbidity Index; DS, Durie-Salmon; ECOG-PS, Eastern Cooperative Oncology Group performance status; eGFR, estimated glomerular filtration rate; FLC, free light chain; IADL, Instrumental Activity of Daily Living; IQR, interquartile range; LAVI, left atrial volume index; LDH, lactate dehydrogenase; LVDD, left ventricular diastolic dysfunction; LVEF, left ventricular ejection fraction; NT-proBNP, N-terminal pro-brain natriuretic peptide; R-ISS, revised International Staging System; TR, tricuspid regurgitation.

^†^*n* = 131 (lower NT-proBNP, *n* = 67; higher NT-proBNP, *n* = 64).

### Clinical characteristics of patients according to NT-proBNP levels: association with LVDD and myeloma-related renal insufficiency

As previously proposed [14], we divided patients into two groups with lower (<300 ng/L) and higher (≥300 ng/L) NT-proBNP levels. The clinical characteristics of patients with lower (*n* = 75) or higher (*n* = 78) NT-proBNP levels are summarized in Table 1. Patients with higher NT-proBNP levels were significantly older; had worse frailty scores; had a higher prevalence of previous histories of arrhythmia and chronic heart failure; had higher beta 2-microglobulin and lactate dehydrogenase levels; and had lower albumin, estimated glomerular filtration rate (eGFR), and hemoglobin levels. Patients with higher NT-proBNP levels also had higher R-ISS stages. However, there were no significant differences between the two groups regarding sex, prevalence of high-risk cytogenetic abnormality (CA) (del(17p), t(4;14), or t(14;16) detected by interphase fluorescence *in situ* hybridization analysis), and treatment, including autologous stem cell transplantation (ASCT).

Patients with higher NT-proBNP levels more frequently had LVDD than patients with lower NT-proBNP levels (67.2% vs. 32.8%, respectively; *P <* 0.001). Furthermore, all diastolic function-related parameters showed associations with NT-proBNP levels, although the difference in septal e’ did not reach statistical significance. However, there was no significant difference in the LV ejection fraction (LVEF) between patients with lower and higher NT-proBNP levels.

We also investigated the association between NT-proBNP levels and myeloma-related renal insufficiency. Patients with higher NT-proBNP levels more frequently had light chain cast nephropathy (41.0% vs. 4.0%, respectively; *P <* 0.001) or hypercalcemia-associated renal insufficiency (17.9% vs. 1.3%, respectively; *P <* 0.001). Such patients also had significantly higher free light chain (FLC) and corrected calcium levels compared to patients with lower NT-proBNP levels. Accordingly, patients with higher NT-proBNP levels more frequently had myeloma-related renal insufficiency (47.4% vs. 5.3%, respectively; *P <* 0.001).

### Survival outcomes according to the NT-proBNP levels

**Figure 1:**
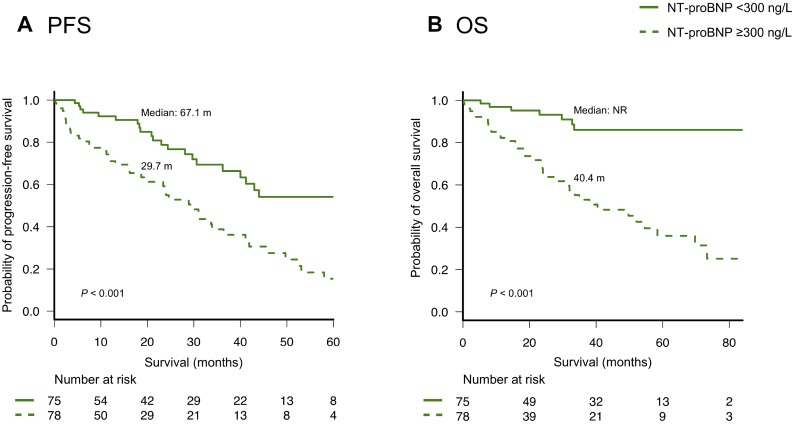
(**A**) Progression-free survival (PFS) and (**B**) overall survival (OS) according to the levels (< or ≥300 ng/L) of N-terminal pro-brain natriuretic peptide (NT-proBNP).

Kaplan-Meier survival curves for progression-free survival (PFS) and overall survival (OS) according to NT-proBNP levels are shown in Figure 1. Patients with higher NT-proBNP levels had both significantly shorter PFS and OS than patients with lower NT-proBNP levels (median PFS: 24.6 and 67.1 months, respectively; *P <* 0.001, and median OS: 40.4 months and not reached, respectively; *P <* 0.001). Patients with higher NT-proBNP levels had significantly shorter OS than patients with lower NT-proBNP levels in both younger (<70 years) or older (≥70 years) groups (Supplementary Figure 1) and in both higher (≥50 ml/min/1.73 m^2^) and lower (<50 ml/min/1.73 m^2^) eGFR groups (Supplementary Figure 2). The NT-proBNP levels also discriminated two groups with different OS among both 43 and 110 patients who received treatment with and without ASCT, respectively (Supplementary Figure 3). Furthermore, there was a sharp discrimination of the OS curves based on the NT-proBNP levels among both patients with R-ISS stage II and stage III (Supplementary Figure 4).

On univariate analysis, age, ECOG-PS, IADL, CCI, NT-proBNP, LVDD, and the R-ISS significantly predicted OS. Despite the relationship between NT-proBNP levels and other clinical factors, NT-proBNP retained its prognostic value for OS in the multivariate analysis (Supplementary Table 2). Notably, LVDD also showed independent prognostic impact for OS.

### Validation and modification of Mayo Clinic's new frailty system

**Figure 2:**
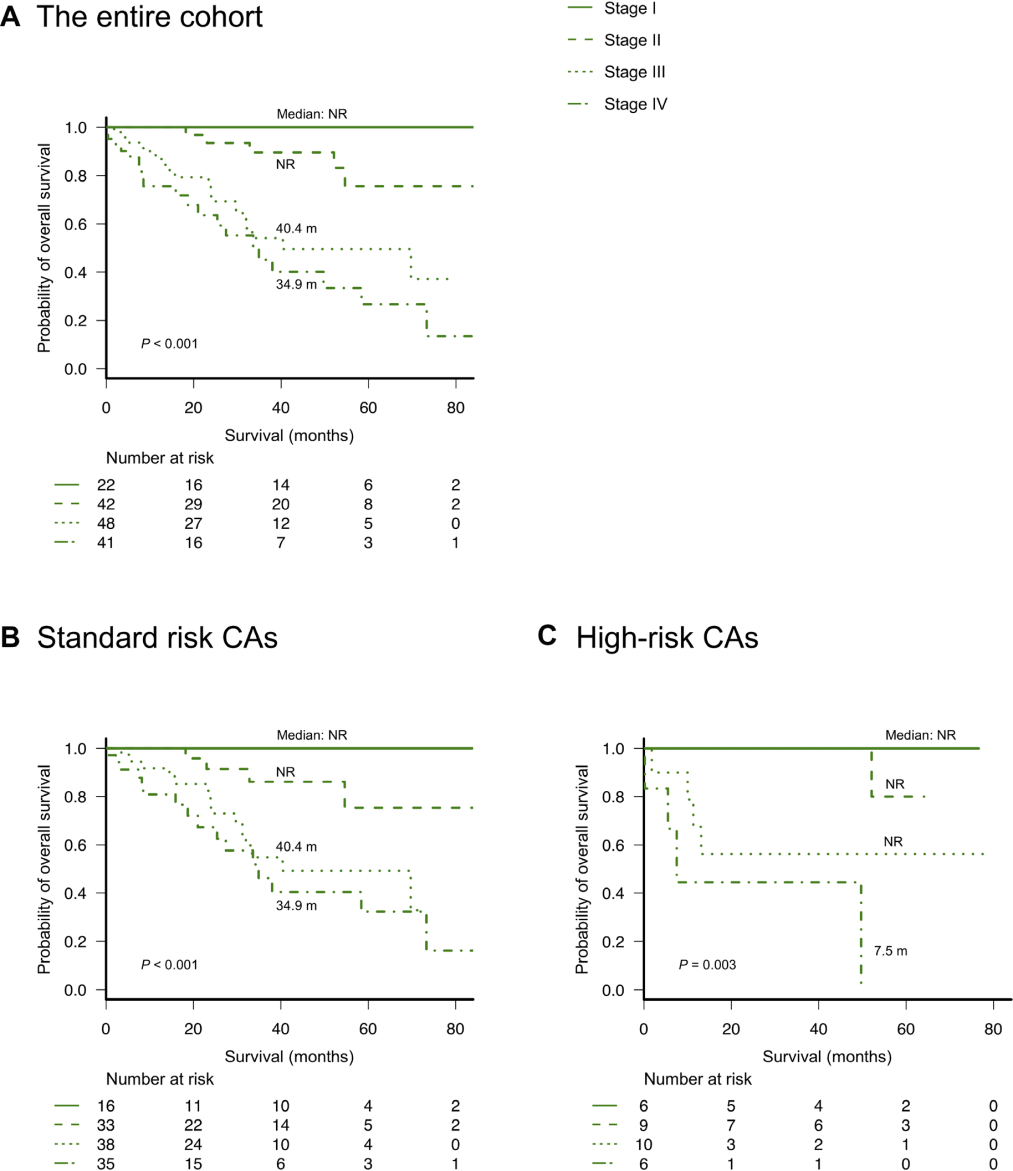
Overall survival according to Mayo Clinic’s new frailty system based on age ≥70 years, ECOG-PS ≥2, and NT-proBNP ≥300 ng/L (different stages based on a score of 0–3 points, respectively) (**A**) overall survival according to the frailty system in the entire cohort, (**B**) overall survival according to the frailty system in patients without high-risk cytogenetic abnormalities, and (**C**) overall survival according to the frailty system in patients with high-risk cytogenetic abnormalities [del(17p), t(4;14), or t(14;16) detected by fluorescence *in situ* hybridization analysis].

**Figure 3:**
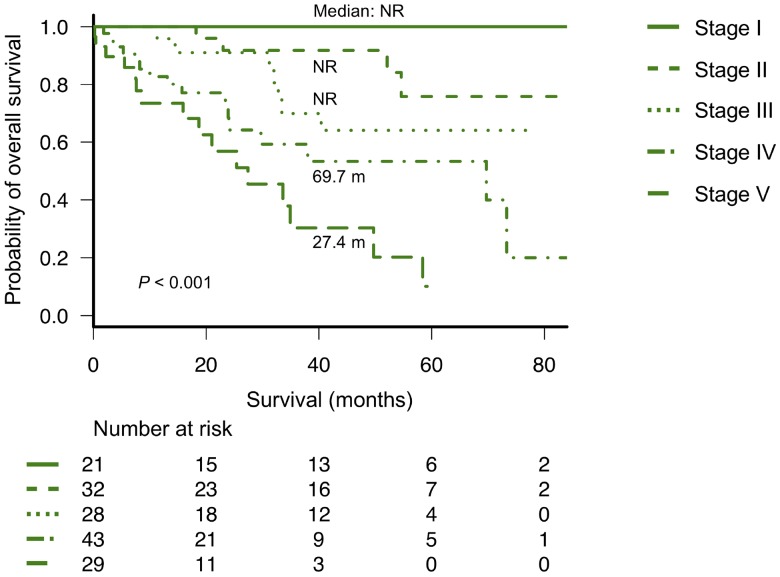
Overall survival according to the modified new frailty system based on age ≥70 years, ECOG-PS ≥2, IADL ≤4, and NT-proBNP ≥300 ng/L (different stages based on a score of 0 to 4 points) ECOG-PS, Eastern Cooperative Oncology Group performance status; IADL, Instrumental Activity of Daily Living; NT-proBNP, N-terminal pro-brain natriuretic peptide.

As described in a previous report from Mayo Clinic [14], we divided patients into four groups using Mayo Clinic's new frailty system that incorporated three risk factors: age ≥70 years, ECOG-PS ≥2, and NT-proBNP levels ≥300 ng/L. This frailty system sharply discriminated four different groups in the entire cohort as well as in patients with or without high-risk CAs, as observed in Mayo Clinic's previous study (Figure 2). We further analyzed the value of this frailty system by adjusting for the IADL score, which was not evaluated in the previous study conducted by Mayo Clinic. Notably, the IADL was still an independent prognostic parameter for OS even after adjusting for Mayo Clinic's new frailty system and the R-ISS (Table 2A). Therefore, we modified Mayo Clinic's new frailty system by incorporating the IADL score; this modified, new frailty system included age ≥70 years, ECOG-PS ≥2, IADL ≤4, and NT-proBNP levels ≥300 ng/L, with each parameter scoring 1 point, thus creating a staging system ranging from I to V. Notably, this modified frailty system showed excellent discrimination of the OS curves across the five groups (Figure 3). Furthermore, this modified frailty system remained an independent predictor of the R-ISS in multivariate analysis (Table 2B). Receiver operating characteristic (ROC) curves were developed for a basic model that consists of the variables used in Mayo Clinic's new frailty system and a model consisting of this basic model + IADL for 5-year OS (Supplementary Figure 5). Incorporation of IADL into the basic Mayo Clinic's frailty model resulted in an increase in area under curve (AUC) from 0.80 (95% confidence interval [CI]; 0.68–0.92) to 0.87 (95% CI; 0.78–0.96), although it did not reach statistical significance (*P* = 0.10). Calibration analysis showed an agreement between the observed risk and the risk predicted by Mayo Clinic's frailty system model and our modified, new frailty system model (*P* = 0.64 and *P* = 0.15, respectively). Continuous net reclassification improvement (NRI) and integrated discrimination improvement (IDI) were significant (NRI 1.093 [95% CI; 0.564–1.621], *P <* 0.001; IDI 0.070 [95% CI; 0.001–0.138], *P* = 0.042).

Table 2Multivariate Cox regression analyses predicting overall survival

**Table:** 

A		
Variables	HR (95% CI)	*P*
IADL, ≤4	2.25 (1.06–4.78)	0.035
CCI, ≥2	0.67 (0.32–1.40)	0.29
Mayo Clinic's new frailty system (ECOG-PS-Age-NT-proBNP)	2.04 (1.35–3.10)	0.001
R-ISS	2.00 (1.09–3.67)	0.024

**Table:** 

B		
Variables	HR (95% CI)	*P*
Modified new frailty system (ECOG-PS-IADL-Age-NT-proBNP)	1.62 (1.28–2.06)	<0.001
R-ISS	1.88 (1.05–3.36)	0.032

Abbreviations: CCI, Charlson Comorbidity Index; CI, confidence interval; ECOG-PS, Eastern Cooperative Oncology Group performance status; HR, hazard ratio; IADL, Instrumental Activity of Daily Living; NT-proBNP, N-terminal pro-brain natriuretic peptide; R-ISS, revised International Staging System.

### Changes in NT-proBNP levels on follow-up tests according to the presence of LVDD or myeloma-related renal insufficiency

**Figure 4:**
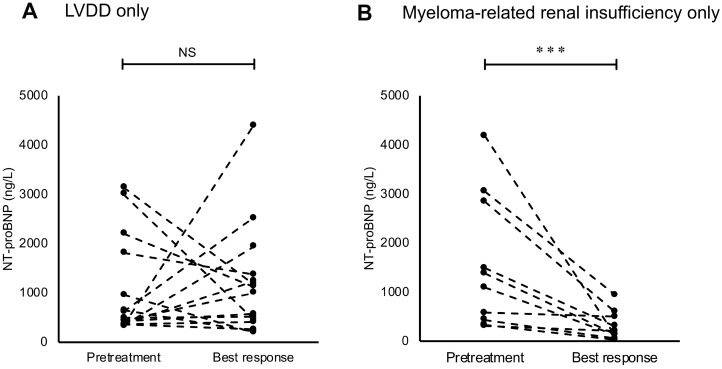
Changes in the levels of N-terminal pro-brain natriuretic peptide (NT-proBNP) on follow-up tests at best response in (**A**) patients with only left ventricular diastolic dysfunction (LVDD) and (**B**) patients with only myeloma-related renal insufficiency. Asterisks (^*^) indicate statistical significance: ^***^P < 0.001, NS: not significant.

Figure 4A and 4B show the NT-proBNP levels on pretreatment tests and follow-up tests at best response in patients with only LVDD (*n* = 17) and those with only myeloma-related renal insufficiency (*n* = 12) who had higher NT-proBNP levels on pretreatment tests. With a median time interval of 3.6 (IQR: 2.5–6.4) months, NT-proBNP levels in patients with only LVDD showed no significant change at best response compared with the pretreatment test levels. In 5 (29.4%) of these patients, NT-proBNP levels decreased by half; these patients had no difference in specific clinical characteristics compared to that of the remaining patients. Conversely, NT-proBNP levels in patients with only myeloma-related renal insufficiency showed a significant reduction from the pretreatment levels with a median time interval of 2.6 (IQR: 1.4–3.8) months (*P <* 0.001).

## DISCUSSION

In the present study, we investigated the relationship between NT-proBNP levels and clinical parameters, including frailty scores, cardiac (including LV diastolic function), and renal function, in real-world patients with symptomatic MM with no concomitant light chain (AL) amyloidosis. We further sought to validate the prognostic value of the NT-proBNP levels as well as the new frailty system suggested by the Mayo Clinic study [14].

NT-proBNP has shown robust prognostic relevance in AL amyloidosis [18] and has thus been incorporated into major staging systems for diagnosis [19]. In addition, previous studies in AL amyloidosis have demonstrated that NT-proBNP levels differ in patients with or without cardiac involvement characterized by ventricular wall thickness. However, its prognostic performance and cardiac disorder patterns shown by NT-proBNP, which may have a negative prognostic impact, have not been fully elucidated in symptomatic MM. The results of this study suggested that NT-proBNP had prognostic significance even in symptomatic MM, especially for OS. These reproducible results observed across different disease entities might be related to the potential capability of NT-proBNP to reflect both cardiac and renal dysfunctions with great sensitivity on the basis of unclarified molecular mechanisms in patients whose hearts and kidneys are concurrently affected.

Our data revealed that NT-proBNP levels were associated with the incidence of LVDD, as previously described in non-cancer-bearing patients [20], but not with conventional LVEF in MM. Furthermore, the presence of LVDD was detected as an independent predictor for OS. The prognostic implications of LVDD may be partly related to its association with tolerability against cardiotoxicity of anti-myeloma agents such as proteasome inhibitors (PIs) and cytotoxic agents including high-dose chemotherapy for ASCT recipients [21, 22]. In particular, PIs remarkably improved survival in MM [23], despite its potential cardiotoxicity [24]. Therefore, novel agent-based treatments might have made the prognostic relevance of subclinical cardiac dysfunctions greater, relative to that in the pre-novel agent era. Additionally, the negative influence of LVDD has been described even in the context of the natural history of healthy subjects [25]. Thus, we believe that the assessment of LV diastolic function will be of utmost importance in the evaluation of host factors for the prognostication of MM patients who are treated with or without PIs.

We also revealed that NT-proBNP was associated with myeloma-related renal insufficiency based on the high frequency of light chain cast nephropathy and hypercalcemia-associated renal insufficiency among patients with higher NT-proBNP levels. Several studies have shown that high levels of baseline serum calcium and involved FLC are associated with disease aggressiveness and unfavorable outcomes in symptomatic myeloma [26–28]. In contrast to LVDD patients, NT-proBNP levels in patients with myeloma-related renal insufficiency significantly decreased at disease remission. Accordingly, NT-proBNP possibly reflects both host and disease factors and could be used as a comprehensive marker for the prognostic assessment of MM. Indeed, NT-proBNP levels discriminated between patients with significantly different survivals even in both younger and older patients or in patients with or without decreased renal functions. Furthermore, multivariate analysis identified NT-proBNP as an independent prognostic predictor.

In comparison to the cohort in the previous report from Mayo Clinic [14], our cohort included considerably older patients (median age: 65 vs. 74 years) reflecting the real-world Japanese population. As a result, the absolute NT-proBNP levels as well as the frailty scores were greater in our cohort [29]. However, Mayo Clinic's new frailty system demonstrated excellent performance in our cohort despite this clear age difference. These results are of particular interest because the peculiar distribution of patients’ age observed in our study may exemplify the future situations in other developed Western countries approaching a period of super-aging such as that currently observed in Japan. Moreover, we found a prognostic value for the IADL score, which was not evaluated in Mayo Clinic's study despite its established utility in the geriatric assessment [8], independent of the new frailty system; the incorporation of the IADL could further improve this frailty system.

The present study has several limitations, including its retrospective nature, heterogeneous treatments, and relatively small sample size. Despite these limitations, this study highlighted the association between NT-proBNP and LV diastolic function or myeloma-related renal insufficiency and a promising prognostic potential of NT-proBNP per se or as a part of the newer frailty systems in patients with symptomatic myeloma.

In conclusion, our findings revealed that the baseline NT-proBNP levels were associated with both LVDD (as a host risk factor) and myeloma-related renal insufficiency (resulting from disease aggressiveness). In addition, this is the first validation study of Mayo Clinic's new frailty system. Our results validated the robust prognostic relevance of NT-proBNP and the usefulness of Mayo Clinic's new frailty system in a real-world context. Moreover, our modification further improved Mayo Clinic's system by newly incorporating the IADL score. Further longitudinal studies are warranted to validate our results and establish the value of NT-proBNP and these frailty systems for the routine assessment of symptomatic MM in the era of novel agents.

## MATERIALS AND METHODS

### Study design and patients

This study retrospectively analyzed data of 153 consecutive newly diagnosed symptomatic MM patients treated with chemotherapy between April 2011 and March 2018 at Kameda Medical Center, Chiba, Japan. We routinely perform bone marrow and subcutaneous adipose tissue biopsy; further, we perform biopsies of targeted organs when suspected for the detection of light chain AL amyloidosis in all patients. Patients with pathologically proven involvement with AL amyloidosis were excluded from the analysis. The diagnoses and treatment responses were evaluated using the IMWG criteria [30, 31]. We included only patients who had been treated with novel agents (e.g., immunomodulatory agents or PIs). As part of the initial evaluations of patients at admission, ECOG-PS, IADL score, and CCI were routinely calculated. Pretreatment levels of NT-proBNP were routinely measured on sera using standard commercially available assays. Serum FLC levels were quantified in all patients with the use of the Freelite assay (The Binding Site, UK) on a Dade-Behring BN II Nephelometer. Serum calcium levels were corrected for hypoalbuminemia appropriately. The presence of LVDD was determined individually according to the 2016 American Society of Echocardiography and European Association of Cardiovascular Imaging guidelines [32], which is briefly described in Doc. S1. The diagnoses of renal insufficiency, light chain cast nephropathy, and hypercalcemia were made according to the updated IMWG criteria [30], which is also briefly described in Doc. S1. Myeloma-related renal insufficiency included light chain cast nephropathy and hypercalcemia-associated renal insufficiency in this study. Written informed consent was obtained from all patients or their families. The study was conducted according to the Declaration of Helsinki and was approved by the review board of Kameda Medical Center.

### Statistical analysis

The relationship between baseline characteristics and NT-proBNP levels were compared using one-way analysis of variance, Kruskal-Wallis tests, Wilcoxon signed rank tests, or chi-squared tests, as appropriate. PFS was defined as the time from the date of diagnosis to the date of confirmation of the first disease progression or death from any cause, whichever occurred first. OS was defined as the time from the diagnosis to the date of death from any cause or last follow-up [4]. The probability of PFS and OS was determined using the Kaplan-Meier method and compared using the log-rank test. The prognostic impact of NT-proBNP levels as well as the frailty systems was evaluated using multivariate Cox proportional-hazards analyses. In the multivariate analyses, adjustment was performed for age, other frailty scores, and R-ISS, as previously described in Mayo Clinic's study [14].

To assess the effect of the addition of IADL data on Mayo Clinic's frailty system, we tested its discrimination and reclassification capabilities by the ROC curve and AUC, NRI, and IDI. We constructed a ROC curve for two logistic regression models. The basic model was constructed with variables which comprise Mayo Clinic's frailty system (age, ECOG-PS, and NT-proBNP), while the basic model + IADL model was constructed with the basic model plus IADL. Difference in the AUC was compared using DeLong's approach [33]. Continuous NRI and IDI were also determined to evaluate the additive prognostic value of IADL [34]. The goodness-of-fit test statistics were also calculated to assess the calibration for the prediction models using the Hosmer-Lemeshow method [35]. A two-tailed *P* value <0.05 was considered statistically significant. Statistical analysis was performed using STATA version 15.1 (Stata corp., College Station, TX, USA) for the ROC analyses and R version 3.1.2 (R Foundation for Statistical Computing, Vienna, Austria) for the remaining analyses.

## SUPPLEMENTARY MATERIALS FIGURES AND TABLES



## Abbreviations

ADL: activity of daily living
AL amyloidosis: light chain amyloidosis
AUC: area under the curve
ASCT: autologous stem cell transplantation
CA: cytogenetic abnormality
CCI: Charlson Comorbidity Index
CI: confidence interval
ECOG-PS: Eastern Cooperative Oncology Group performance status
eGFR: estimated glomerular filtration rate
FLC: free light chain
IADL: Instrumental Activity of Daily Living
IMWG: International Myeloma Working Group
IQR: interquartile range
ISS: International Staging System
LV: left ventricular
LVDD: left ventricular diastolic dysfunction
LVEF: left ventricular ejection fraction
MM: multiple myeloma
NT-proBNP: N-terminal pro-brain natriuretic peptide
OS: overall survival
PFS: progression-free survival
PI: proteasome inhibitor
R-ISS: Revised International Staging System
ROC: receiver operating characteristic.
