# Supplementary material for: N-terminal pro-brain natriuretic peptide reflects both left ventricular diastolic dysfunction and myeloma-related renal insufficiency and robustly predicts mortality in patients with symptomatic multiple myeloma
Source: Oncotarget. 2019 Feb 1;10(10):1160–70. doi: 10.18632/oncotarget.26647 (PMC6383683; doi:10.18632/oncotarget.26647)
Supplement: Supplementary file 1 [file oncotarget-10-1160-s001.pdf]

N-terminal pro-brain natriuretic peptide reflects both left ventricular diastolic dysfunction and myeloma-related renal insufficiency and robustly predicts mortality in patients with symptomatic multiple myeloma

SUPPLEMENTARY MATERIALS

Definition of the presence of left ventricular diastolic dysfunction, renal insufficiency, and light chain cast nephropathy

We determined the presence or absence of left ventricular diastolic dysfunction (LVDD) individually according to the 2016 American Society of Echocardiography and European Association of Cardiovascular Imaging guidelines [1]. Briefly, the following criteria were initially checked: E/e' (the ratio of early mitral inflow velocity [E] to mitral annular early diastolic velocity [e']) >14, left atrial volume index (LAVI) >34 ml/m^2, septal e' velocity <7 cm/s, and tricuspid regurgitation (TR) velocity >2.8 m/s. If >50% of measurable parameters meet the criteria above, then LVDD was deemed present in this study.

According to the updated IMWG criteria [2], renal insufficiency was defined as an estimated creatinine clearance <40 ml/min or serum creatinine >2 mg/dl while light chain cast nephropathy was diagnosed based on the presence of renal insufficiency with typical histological changes for definitive diagnosis or high involved free light chain levels of >1500 mg/L for presumptive diagnosis. Hypercalcemia was defined as serum calcium >1 mg/dL higher than the upper limit of normal or >11 mg/dL. Hypercalcemia-associated renal insufficiency was deemed present when renal insufficiency was present with concomitant hypercalcemia.

REFERENCES

1. Nagueh SF, Smiseth OA, Appleton CP, Byrd BF, Dokainish H, Edvardsen T, Flachskampf FA, Gillebert TC, Klein AL, Lancellotti P, Marino P, Oh JK, Popescu BA, et al. Recommendations for the evaluation of left ventricular diastolic function by echocardiography: An update from the American Society of Echocardiography and the European Association of Cardiovascular Imaging. J Am Soc Echocardiogr. 2016; 29:277–314.
2. Rajkumar SV, Dimopoulos MA, Palumbo A, Blade J, Merlini G, Mateos MV, Kumar S, Hillengass J, Kastritis E, Richardson P, Landgren O, Paiva B, Dispenzieri A, et al. International Myeloma Working Group updated criteria for the diagnosis of multiple myeloma. Lancet Oncol. 2014; 15:e538–e548.

Supplementary Table 1: Comparison of baseline clinical characteristics between patients with and without echocardiography data for left ventricular diastolic function

Clinical factors	LV diastolic function data		*P*
	Absent *n* = 22	Present *n* = 131	
**Background and/or frailty parameters**			
Age, years (median [IQR])	72.3 [63.5, 79.2]	74.4 [66.9, 80.9]	0.55
Sex, male (%)	14 (63.6)	62 (47.3)	0.17
ECOG-PS, ≥ 2 (%)	13 (59.1)	70 (53.4)	0.65
IADL, ≤ 4 (%)	9 (40.9)	63 (48.1)	0.64
CCI, ≥ 2 (%)	14 (63.6)	60 (45.8)	0.16
**Past medical histories**			
Coronary artery disease (%)	0 (0.0)	11 (8.4)	0.36
Arrhythmia (%)	0 (0.0)	10 (7.6)	0.35
Chronic heart failure (%)	0 (0.0)	15 (11.5)	0.13
Hypertension (%)	10 (45.5)	76 (58.0)	0.35
Diabetes mellitus (%)	3 (13.6)	31 (23.7)	0.41
**Myeloma-related factors**			
Heavy chain type, IgG (%)	11 (50.0)	68 (51.9)	1.0
Albumin, g/dL (median [IQR])	3.4 [2.3, 4.0]	3.4 [2.9, 3.9]	0.40
Beta 2-microglobulin, mg/L (median [IQR])	6.7 [2.9, 9.7]	4.5 [2.8, 8.0]	0.22
eGFR, mL/min/1.73 m2 (median [IQR])	40.5 [11.9, 64.2]	51.6 [31.1, 69.9]	0.098
Corrected calcium, mg/dL (median [IQR])	9.6 [9.4, 10.2]	9.7 [9.2, 10.4]	0.71
Hypercalcemia-associated renal insufficiency (%)	1 (4.5)	14 (10.7)	0.69
Involved FLC, mg/L (median [IQR])	1390 [489, 3920]	480 [121, 3137]	0.12
Light chain cast nephropathy (%)	7 (31.8)	28 (21.4)	0.28
Myeloma-related renal insufficiency (%)	7 (31.8)	34 (26.0)	0.83
LDH, high (%)	8 (36.4)	33 (25.2)	0.30
High-risk CA (%)	3 (13.6)	28 (21.4)	0.56
R-ISS, ≥ stage II (%)	6 (27.3)	30 (22.9)	0.36
DS system, stage III (%)	17 (77.3)	76 (58.0)	0.10
Bortezomib use (%)	22 (100.0)	126 (96.2)	1.0
Lenalidomide use (%)	20 (90.9)	116 (88.5)	1.0
ASCT recipients (%)	7 (31.8)	36 (27.5)	0.79
**Cardiological parameters**			
NT-proBNP, ng/L (median [IQR])	403 [197, 1061]	279 [122, 1009]	0.30
LVEF, % (median [IQR])	72.0 [70.5, 73.2]	69.5 [66.0, 73.0]	0.14

Abbreviations: ASCT, autologous stem cell transplantation; CA, cytogenetic abnormality; CCI, Charlson Comorbidity Index; DS, Durie-Salmon; ECOG-PS, Eastern Cooperative Oncology Group performance status; eGFR, estimated glomerular filtration rate; FLC, free light chain; IADL, Instrumental Activity of Daily Living; IQR, interquartile range; LDH, lactate dehydrogenase; LV, left ventricular; LVEF, left ventricular ejection fraction; NT-proBNP, N-terminal pro-brain natriuretic peptide; R-ISS, revised International Staging System.

Supplementary Table 2: Univariate and multivariate Cox regression for overall survival

Variables	Univariate Cox regression		Multivariate Cox regression	
	**HR (95% CI)**	***P***	**HR (95% CI)**	***P***
Age, ≥70 years	3.27 (1.55–6.93)	0.002	1.52 (0.66–3.53)	0.33
ECOG-PS, ≥2	2.91 (1.49–5.68)	0.002	1.73 (0.86–3.47)	0.12
IADL, ≤4	4.02 (2.06–7.84)	<0.001	2.46 (1.11–5.46)	0.027
CCI, ≥2	2.00 (1.07–3.71)	0.029	0.58 (0.27–1.23)	0.15
NT-proBNP, ≥300 ng/L	6.06 (2.69–13.64)	<0.001	2.89 (1.04–8.06)	0.042
LVDD				
Absent	1 (Reference)		1 (Reference)	
Present	2.83 (1.51–5.31)	0.001	2.09 (1.07–4.08)	0.031
R-ISS	2.86 (1.68–4.88)	<0.001	1.85 (0.93–3.69)	0.079

Abbreviations: CCI; Charlson Comorbidity Index, CI; confidence interval, ECOG-PS; Eastern Cooperative Oncology Group performance status, HR; hazard ratio, IADL; Instrumental Activity of Daily Living, LVDD; left ventricular diastolic dysfunction, NT-proBNP; N-terminal pro-brain natriuretic peptide, R-ISS; revised International Staging System.

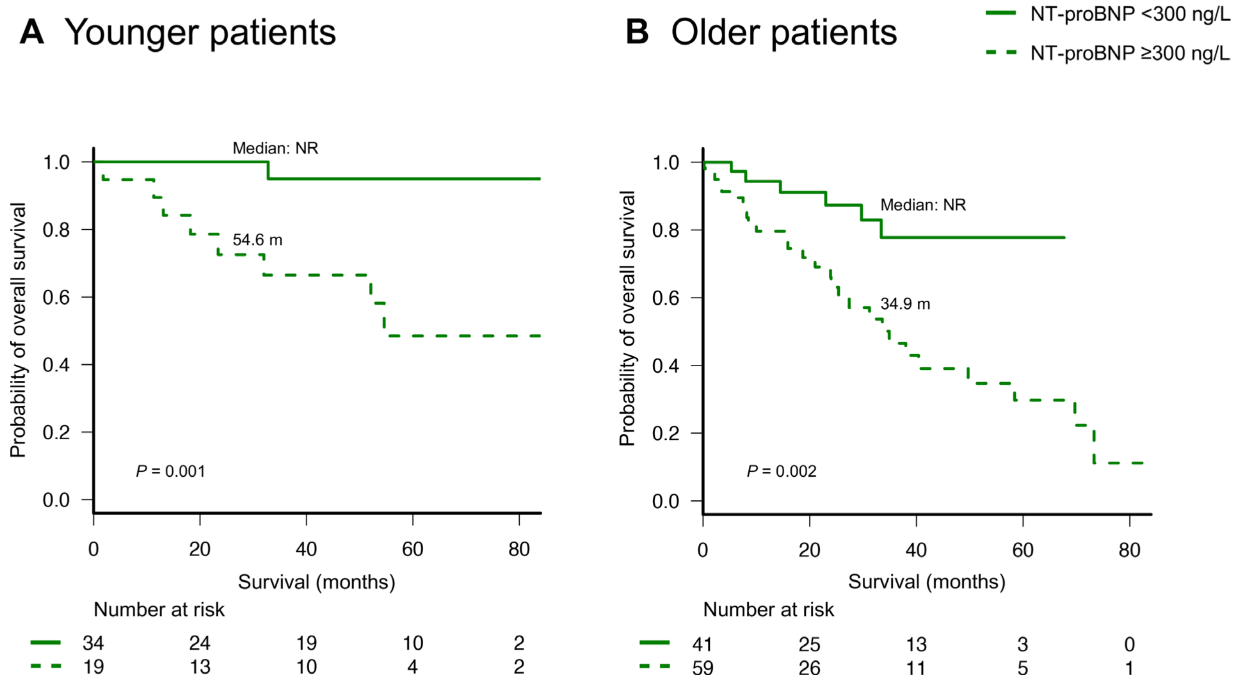

Supplementary Figure 1: Overall survival (OS) according to the levels (< or ≥300 ng/L) of N-terminal pro-brain natriuretic peptide (NT-proBNP) in (**A**) younger (<70 years) or (**B**) older (≥70 years) patients.

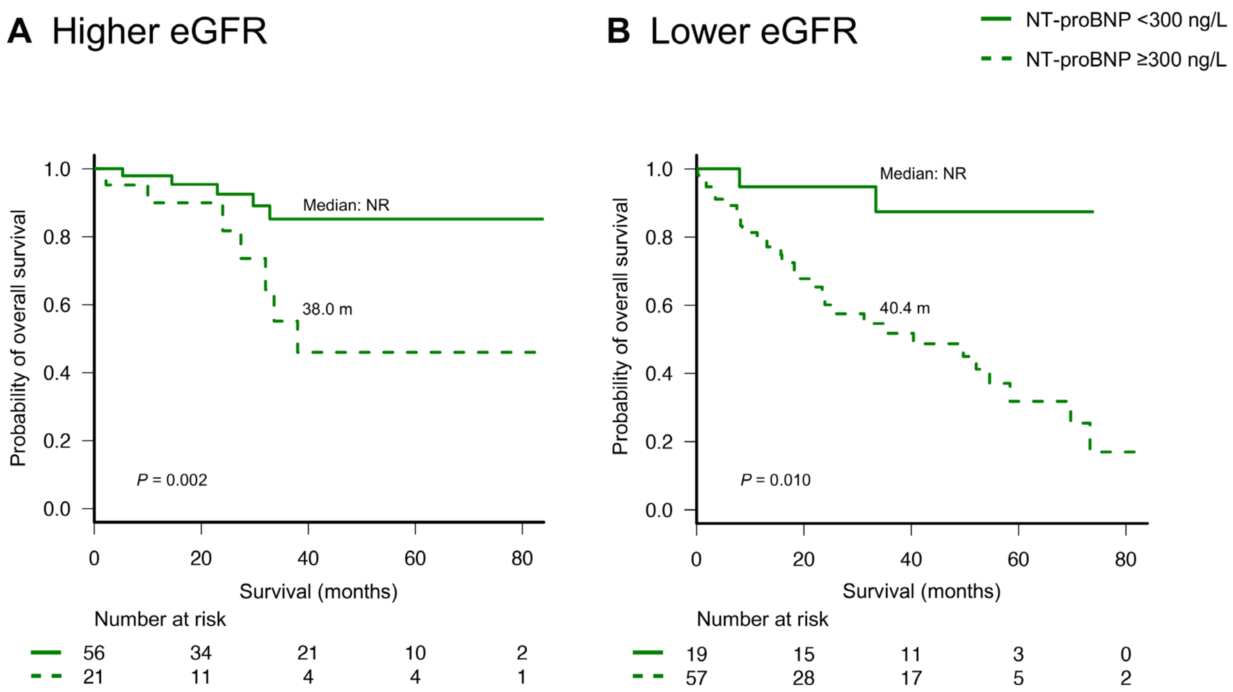

Supplementary Figure 2: Overall survival (OS) according to the levels (< or ≥300 ng/L) of N-terminal pro-brain natriuretic peptide (NT-proBNP) in patients with (**A**) higher or (**B**) lower estimated glomerular filtration rate (GFR) (<50 ml/min/1.73 m^2).

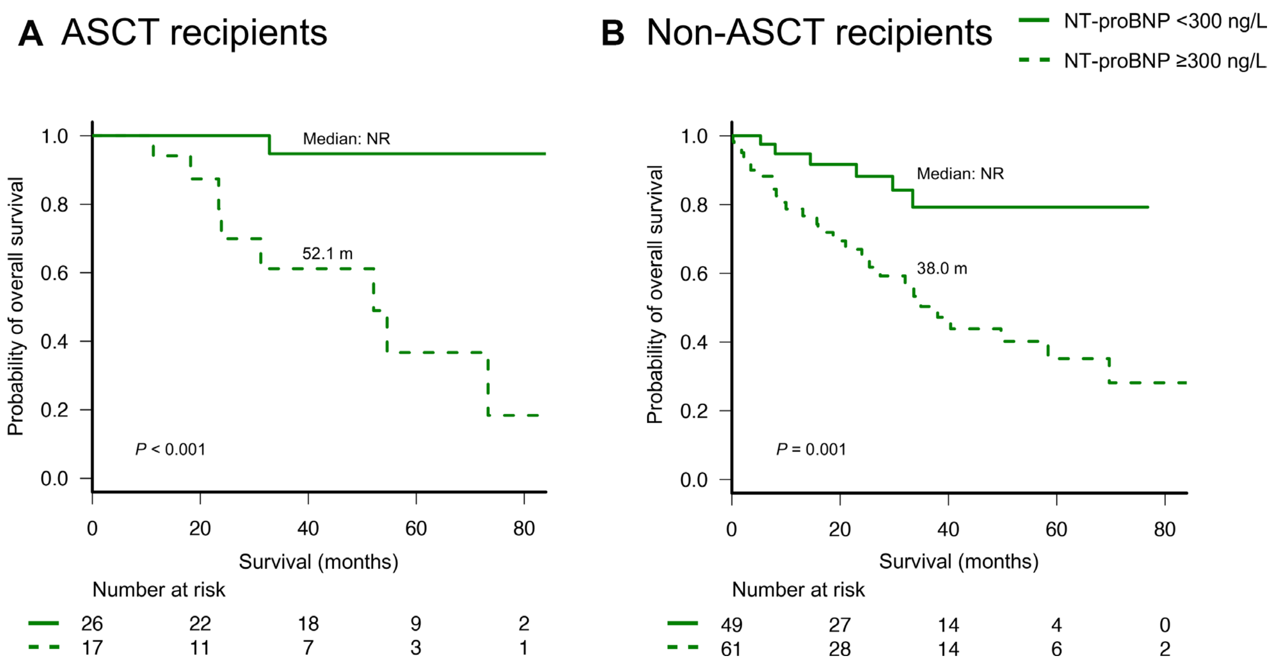

Supplementary Figure 3: Overall survival (OS) according to the levels (< or ≥300 ng/L) of N-terminal pro-brain natriuretic peptide (NT-proBNP) in patients who received treatment (**A**) with or (**B**) without autologous stem cell transplantation.

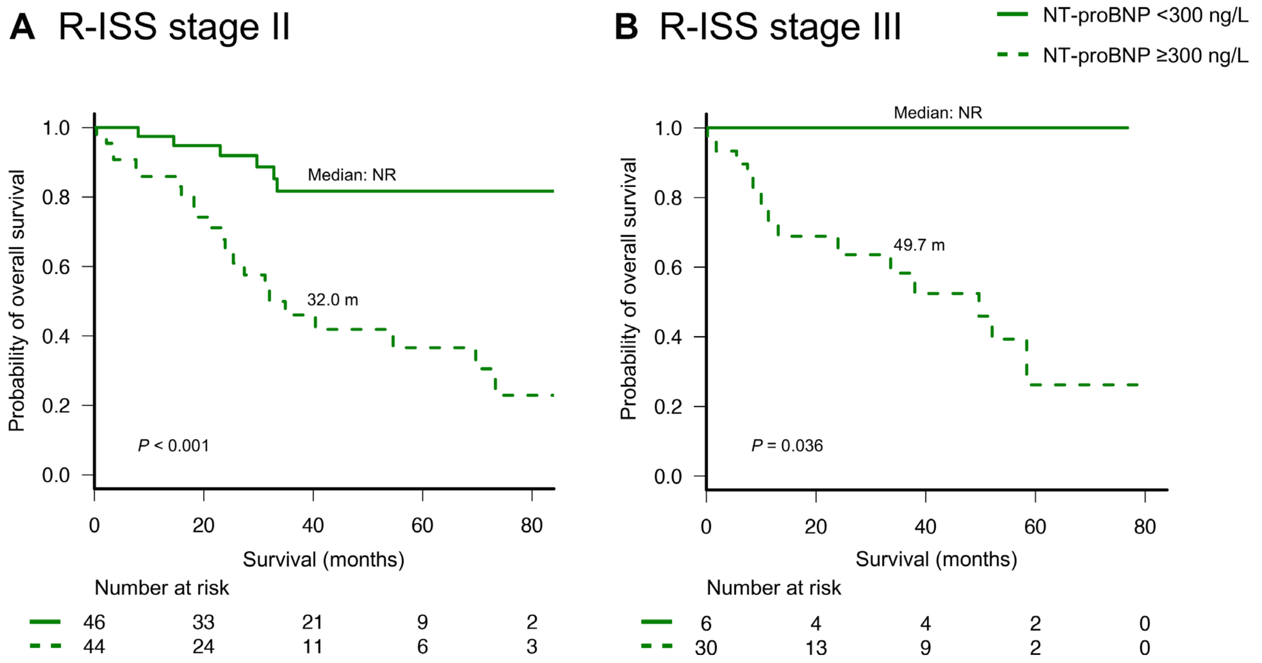

Supplementary Figure 4: Overall survival (OS) according to the levels (< or ≥300 ng/L) of N-terminal pro-brain natriuretic peptide (NT-proBNP) in patients with revised International Staging System (R-ISS) (**A**) stage II or (**B**) stage III.

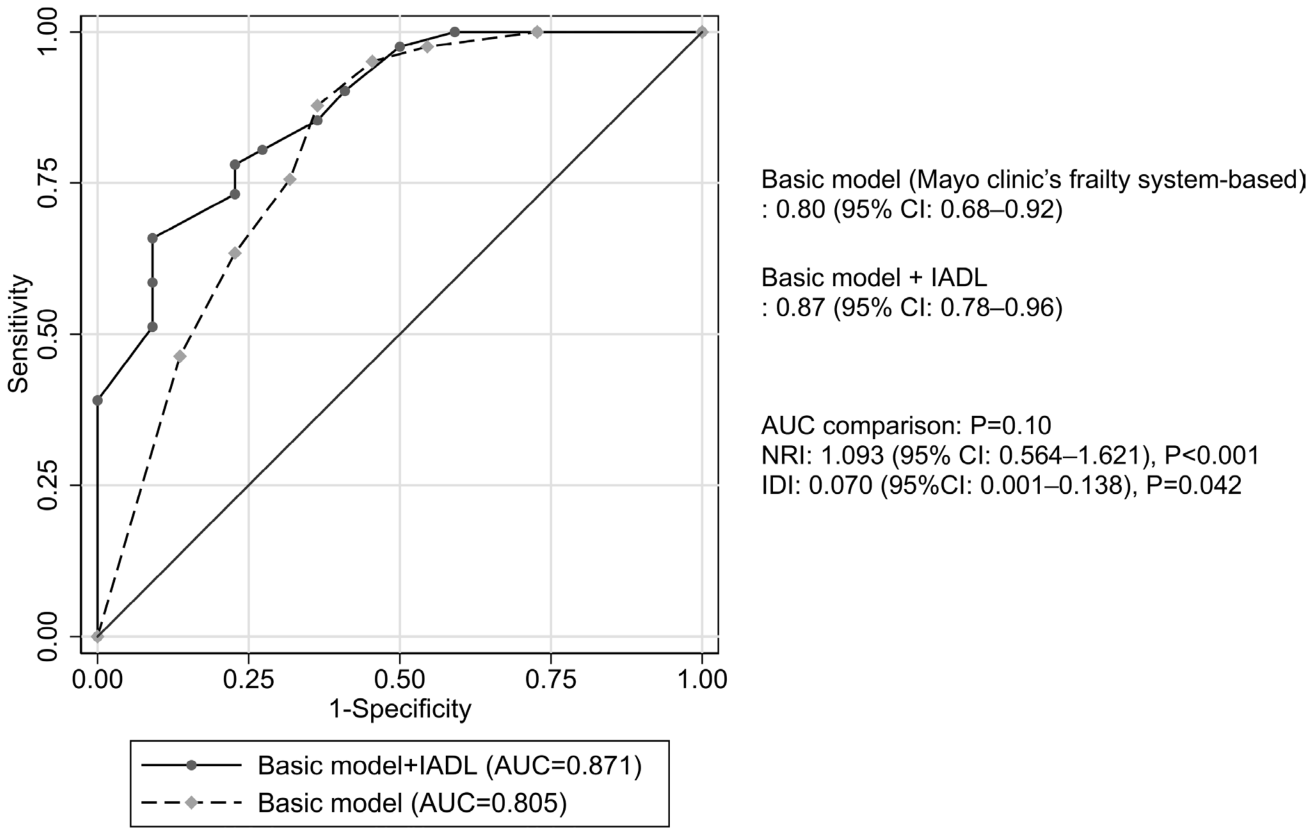

Supplementary Figure 5: Incremental accuracy of adding the instrumental activity of daily living (IADL) on Mayo Clinic's new frailty system model on overall survival.
